# Noninferior Immunogenicity and Consistent Safety of Respiratory Syncytial Virus Prefusion F Protein Vaccine in Adults 50–59 Years Compared to ≥60 Years of Age

**DOI:** 10.1093/cid/ciae364

**Published:** 2024-08-05

**Authors:** Murdo Ferguson, Tino F Schwarz, Sebastián A Núñez, Juan Rodríguez-García, Marek Mital, Carlos Zala, Bernhard Schmitt, Nicole Toursarkissian, Dolores Ochoa Mazarro, Josef Großkopf, Christine Voors-Pette, Hemalini Mehta, Hiwot Amare Hailemariam, Magali de Heusch, Bruno Salaun, Silvia Damaso, Marie-Pierre David, Dominique Descamps, Judith Hill, Corinne Vandermeulen, Veronica Hulstrøm, the RSV OA=ADJ-018 Study Group, Khalid S Abd-Elaziz, Mark S Adams, Agnieszka Barts, Kevin Cannon, Matthew Davis, Sonia de las Fuentes Galán, Marta de los Ríos Rodríguez, Maria Cristina De Salvo, Lauren DeGregoria, Víctor del Campo Pérez, Torsten Drescher, Rebecca Dunsmoor-Su, Peter Dzongowski, Jose Ma Echave-Sustaeta, Tamara Julia Eckermann, Ashley E Fuller, Jaume Garí Parera, Jean Sebastien Gauthier, Steven Geller, Wayne Ghesquiere, Antonio Gonzalez, Patricia González Cediel, Anton Grasch, Laura L Helman, Susan Hernandez, María Herranz Urbasos, Nicolas Itcovici, Terry Klein, Jorge Labrador Gómez, Antonio Lalueza Blanco, Ryan Leblanc, Matthias Luttermann, Kristen Marks, Cristina Masuet-Aumatell, Leonie Möckesch, Tamara Michelle Moreno Silva, Silvia Narejos Perez, Robert J Noveck, Jérôme C Oude Nijhuis, Jean-Sebastien Paquette, Bonavuth Pek, Georg Plassmann, Robert Pritt, Mireia Puig Palma, Claudio Rocha-Calderon, Paule Royer, David Shu, Ying Tung Sia, Angelika Sieber, Todd Simmons, Leslie Sinclair, William B Smith, Joseph Soufer, Ana Suarez Simón, Genoveva Vilardell Rifa, María Teresa Vilella Moreno, Ulrich Weber, Alba María Yañez de la Higuera, Pedro Ylisastigui

**Affiliations:** Colchester Research Group, Truro, Canada; Institute of Laboratory Medicine and Vaccination Centre, Klinikum Würzburg Mitte, Campus Juliusspital, Würzburg Mitte, Würzburg, Germany; Infectología, Centro Medico Maffei, Buenos Aires, Argentina; Preventive Medicine Department, Immunocompromised Patient Immunization Unit, Son Espases University Hospital, Palma de Mallorca, Mallorca, Balearic Islands, Spain; Agnieszka Mital Centrum Badan Clinic, Elblag, Poland; Vacunar, Sede Las Cañitas, Buenos Aires, Argentina; Studienzentrum Mainz Mitte, Mainz, Germany; Praxis Dr. Med. Nicole Toursarkissian, Berlin, Germany; Clinical Pharmacology Department, Hospital Universitario De La Princesa, Instituto de Investigación Sanitaria La Princesa, Universidad Autónoma de Madrid, Madrid, Spain; Praxis Dr. Med. Josef Großkopf, Wallerfing, Germany; QPS Netherlands B.V., Groningen, The Netherlands; Clinical Research Institute, Minneapolis, Minnesota, USA; GSK, Wavre, Belgium; GSK, Wavre, Belgium; GSK, Wavre, Belgium; GSK, Wavre, Belgium; GSK, Wavre, Belgium; GSK, Wavre, Belgium; GSK, Wavre, Belgium; GSK, Wavre, Belgium; GSK, Wavre, Belgium

**Keywords:** respiratory syncytial virus, vaccination, humoral immunity, safety, chronic conditions

## Abstract

**Background:**

The adjuvanted respiratory syncytial virus (RSV) prefusion F protein–based vaccine (RSVPreF3 OA) is approved in adults aged ≥60 years. We evaluated RSVPreF3 OA immunogenicity and safety in adults aged 50–59 years without or with increased risk for RSV disease due to specific chronic medical conditions.

**Methods:**

This observer-blind, phase 3, noninferiority trial included adults aged 50–59 years, stratified into 2 subcohorts: those with and those without predefined, stable, chronic medical conditions leading to an increased risk for RSV disease. Participants in both subcohorts were randomized 2:1 to receive RSVPreF3 OA or placebo. A control group of adults aged ≥60 years received RSVPreF3 OA. Primary outcomes were RSV-A and RSV-B neutralization titers (geometric mean titer ratios and sero-response rate differences) 1 month post-vaccination in 50–59-year-olds versus ≥60-year-olds. Cell-mediated immunity and safety were also assessed.

**Results:**

The exposed population included 1152 participants aged 50–59 years and 381 participants aged ≥60 years. RSVPreF3 OA was immunologically noninferior in 50–59-year-olds versus ≥60-year-olds; noninferiority criteria were met for RSV-A and RSV-B neutralization titers in those with and those without increased risk for RSV disease. Frequencies of RSVPreF3-specific polyfunctional CD4+ T cells increased substantially from pre- to 1 month post-vaccination. Most solicited adverse events had mild-to-moderate intensity and were transient. Unsolicited and serious adverse event rates were similar in all groups.

**Conclusions:**

RSVPreF3 OA was immunologically noninferior in 50–59-year-olds compared to ≥60-year-olds, in whom efficacy was previously demonstrated. The safety profile in 50–59-year-olds was consistent with that in ≥60-year-olds.

**Clinical Trial Registration:**

ClinicalTrials.gov: NCT05590403.

## Teaser

The RSVPreF3 OA immune response in 50–59-year-olds (including those at increased risk of RSV disease) was noninferior to that in ≥60-year-olds, in whom efficacy was demonstrated. The vaccine's safety profile in 50–59-year-olds was consistent with that in ≥60-year-olds.

**(See the Editorial Commentary by Branche on pages 1099–101; Major Article by Clark et al. on pages 1088–98.)**

Respiratory syncytial virus (RSV) is an important cause of acute respiratory illness. Older adults and individuals with certain underlying medical conditions (eg, chronic pulmonary and cardiovascular diseases, diabetes, and chronic renal and liver diseases) are at increased risk of severe outcomes for RSV infection compared to younger adults and those without these conditions [1–6].

To help reduce the RSV burden in older adults, an adjuvanted RSV prefusion F protein–based vaccine (RSVPreF3 OA, Arexvy; GSK) was developed. This vaccine had an efficacy of 82.6% (96.95% confidence interval [CI]: 57.9–94.1%) against RSV-related lower respiratory tract disease [RSV-LRTD] and 94.1% (95% CI: 62.4–99.9%) against severe RSV-LRTD over 1 RSV season in individuals aged 60 years and older [7], and had sustained efficacy over 2 seasons [8]. High efficacy was also shown among those aged 60 years and older with 1 or more chronic conditions that increase the risk for RSV disease (94.6% [95% CI: 65.9–99.9%] against RSV-LRTD over 1 season) [7, 9]. RSVPreF3 OA and another RSV prefusion F-based vaccine were the first 2 vaccines approved for the prevention of RSV-LRTD in those aged 60 years and older [10–13].

**Figure 1.**
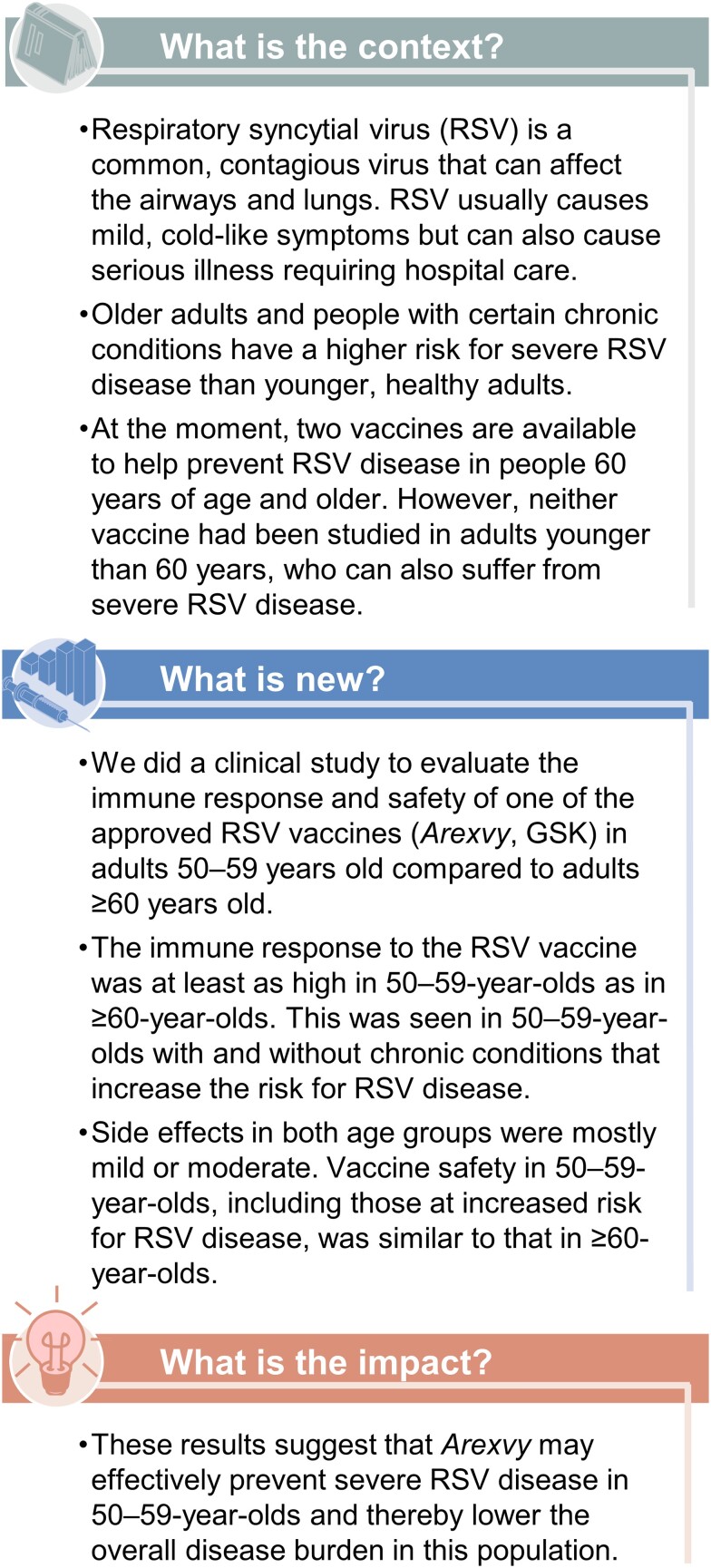
Plain language summary.

Respiratory syncytial virus–associated illness also causes significant morbidity in adults younger than 60 years, especially in those with the aforementioned chronic medical conditions [1–4]. Individuals aged 50–59 years also report a substantial impact of RSV disease on the quality of life, including on productivity, social activities, and emotional and physical functioning [14, 15]. However, to date, no RSV vaccine has been studied or approved in 50–59-year-olds. We therefore evaluated the immunogenicity, reactogenicity, and safety of RSVPreF3 OA in adults aged 50–59 years, including those with chronic medical conditions leading to an increased risk for RSV disease. The study aimed to demonstrate noninferiority of the humoral immune response in this population compared to adults aged 60 years and older, the population for which RSVPreF3 OA efficacy has been demonstrated [7–9]. Figure 1 summarizes the study findings in plain language.

## METHODS

### Study Design, Participants, and Interventions

This ongoing randomized, placebo-controlled, phase 3 study (ClinicalTrials.gov: NCT05590403) takes place in 60 centers in 8 countries (Argentina, Canada, Germany, Japan, The Netherlands, Poland, Spain, and United States). The study includes a cohort with 50–59-year-old participants and a control cohort with participants aged 60 years and older. The cohort with 50–59-year-olds was divided into an at-increased-risk (AIR) and a non-AIR subcohort. The AIR subcohort only included participants diagnosed with at least 1 predefined, stable, chronic medical condition recognized to increase the risk for RSV disease. These conditions were as follows: chronic pulmonary disease (eg, chronic obstructive pulmonary disease, asthma, cystic fibrosis, lung fibrosis, restrictive lung disease, interstitial lung disease, emphysema, and bronchiectasis), chronic cardiovascular disease (ie, chronic heart failure, coronary artery disease, and cardiac arrhythmia), diabetes mellitus types 1 and 2, chronic renal disease, and chronic liver disease. The non-AIR subcohort excluded individuals with these specific conditions but could include people with other stable, non-immunocompromising conditions (eg, hypertension, hypercholesterolemia, or hypothyroidism). Inclusion criteria for the cohort of those aged 60 years and older were aligned with those used in the phase 3 efficacy study [7]; participants aged 60 years and older could have stable, non-immunocompromising, chronic conditions, including those described to increase the risk for RSV disease. A condition was considered stable if there were no changes in treatment or severity in the 3 months before enrollment. Detailed eligibility criteria and enrollment rules are listed in the Supplementary Methods.

**Figure 2.**
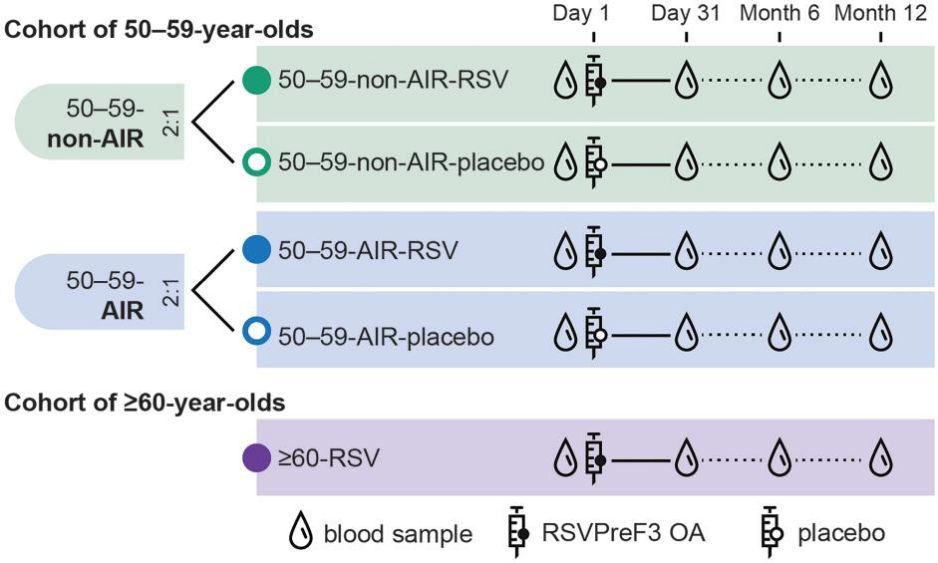
Study design. Abbreviations: 50–59-AIR-placebo, group of 50–59-year-old participants at increased risk for respiratory syncytial virus (RSV) disease who received placebo; 50–59-AIR-RSV, group of 50–59-year-old participants at increased risk for RSV disease who received RSV prefusion F protein–based vaccine (RSVPreF3 OA); 50–59-non-AIR-placebo, group of 50–59-year-old participants without increased risk for RSV disease who received placebo; 50–59-non-AIR-RSV, group of 50–59-year-old participants without increased risk for RSV disease who received RSVPreF3 OA; ≥60-RSV, group of ≥60-year-old participants who received RSVPreF3 OA.

Participants in the non-AIR and AIR subcohorts of 50–59-year-olds were randomized (2:1 in each subcohort) to receive 1 intramuscular dose of RSVPreF3 OA (50–59-non-AIR-RSV and 50–59-AIR-RSV groups) or placebo (50–59-non-AIR-placebo and 50–59-AIR-placebo groups) (Supplementary Methods). Participants in the cohort of participants aged 60 years and older were to receive 1 intramuscular RSVPreF3 OA dose (≥60-RSV group) (Figure 2). The study was observer-blind for the cohort of 50–59-year-olds until the analysis of the primary endpoints (after which it was single-blind); it was open-label for the cohort of participants aged 60 years and older (Supplementary Methods).

The study was conducted according to the ethical principles of the Declaration of Helsinki, Good Clinical Practice guidelines, and applicable regulatory requirements. The protocol and amendment were approved by the relevant ethics committees. A protocol summary is available on https://www.gsk-studyregister.com/en/trial-details/?id=219238. All participants provided written informed consent.

### Objectives

The co-primary objectives were to demonstrate the noninferiority of the humoral immune response to RSVPreF3 OA in 50–59-year-olds, both in the AIR and non-AIR subcohorts, compared to those aged 60 years and older for both RSV-A and RSV-B at 1 month post-vaccination. Secondary objectives included further characterizing the humoral immune response and evaluating cell-mediated immunity, reactogenicity, and safety of RSVPreF3 OA in both age cohorts.

### Immunogenicity Assessments

Blood samples to assess the humoral immune response were to be collected from all participants on day 1 (pre-vaccination), day 31, month 6, and month 12 (Figure 2). Additional blood samples were to be collected from a subset of approximately 350 participants (Supplementary Methods) at the same time points to assess cell-mediated immunity. We report results for the day 1 and day 31 time points.

Serum RSV-A and RSV-B neutralization titers were measured using a neutralization assay and displayed in estimated dilution 60 (ED60) [7]. The frequencies of RSVPreF3-specific CD4+ and CD8+ T cells expressing CD40 ligand (CD40L), 4-1BB, interleukin (IL)-2, tumor necrosis factor-α (TNF-α), interferon-γ (IFN-γ), IL-13, or IL-17 per million of CD4+ or CD8+ T cells were analyzed using flow cytometry after intracellular cytokine staining on peripheral blood mononuclear cells (Supplementary Methods).

### Reactogenicity and Safety Assessments

Participants used paper diaries to record solicited administration-site and systemic adverse events (AEs) starting within 4 days and unsolicited AEs starting within 30 days post-vaccination. Serious AEs (SAEs) and potential immune-mediated diseases (pIMDs) (which were considered as AEs of special interest) were recorded until 6 months post-vaccination. The SAEs and pIMDs deemed to be related to vaccination by the investigator and fatal SAEs were to be recorded until 12 months post-vaccination (study end). Atrial fibrillation, while not considered an identified risk associated with RSVPreF3 OA vaccination, was monitored as an AE of special interest using the aforementioned reporting periods, depending on if the cases were nonserious AEs, SAEs, or fatal/related SAEs.

The intensity of solicited and unsolicited AEs was graded on a scale from 1 (mild) to 3 (severe). Solicited AEs were all considered as causally related to vaccination. For the other AEs, the investigators used available data/resources and their clinical judgment to assess causality.

### Statistical Analyses

The study aimed to enroll approximately 1520 participants: 380 each in the 50–59-non-AIR-RSV and 50–59-AIR-RSV groups, 190 each in the 50–59-non-AIR-placebo and 50–59-AIR-placebo groups, and 380 in the ≥60-RSV group (Supplementary Methods).

Immunogenicity endpoints were analyzed in the per-protocol set (all eligible participants who received RSVPreF3 OA or placebo per protocol, complied with protocol requirements, and had immunogenicity results available pre- and post-vaccination). Safety endpoints were analyzed in the exposed population (all participants who received RSVPreF3 OA or placebo).

To control the type I error at 2.5% (one-sided) for multiplicity, the 4 co-primary objectives were tested according to a predefined sequence, with significance levels as described in the Supplementary Methods. Noninferiority of the humoral immune response to RSVPreF3 OA in 50–59-year-olds without or with increased risk for RSV disease compared to those aged 60 years and older was demonstrated if the upper limits (ULs) of the 2-sided 95% or 97.5% CIs around the adjusted geometric mean titer (GMT) ratios (≥60-RSV/50–59-non-AIR-RSV and ≥60-RSV/50-59-AIR-RSV) were 1.5 or less, and the ULs of the 2-sided 95% or 97.5% CIs around the differences in sero-response rates (≥60-RSV minus 50–59-non-AIR-RSV and ≥60-RSV minus 50–59-AIR-RSV) were 10% or less for both RSV-A and RSV-B at 1 month post-vaccination. Sero-response rate was defined as the percentage of participants with a 4-fold or greater increase in neutralization titers from pre- to 1 month post-vaccination.

Secondary immunogenicity and safety endpoints were analyzed descriptively. The GMTs and mean geometric increases (ie, geometric means of the within-participant ratios of the post-vaccination over the pre-vaccination neutralization titers) were calculated with 95% CIs, and sero-response rates with exact 95% CIs. The frequencies (median, minimum, maximum, interquartile range) of RSVPreF3-specific CD4+ and/or CD8+T cells expressing 2 or more activation markers including 1 or more cytokine among CD40L, 4-1BB, IL-2, TNF-α, IFN-γ, IL-13, and IL-17 (per million of CD4+/CD8+ T cells) were calculated. Percentages of participants with AEs (solicited, unsolicited, SAEs, pIMDs) were calculated with exact 95% CIs.

All analyses were conducted using SAS Life Science Analytics Framework version 5.4 (SAS Institute, Cary, NC, USA).

The data lock point of the reported analyses was 5 September 2023, with a safety data lock of 1 September 2023.

## RESULTS

### Study Participants

**Figure 3.**
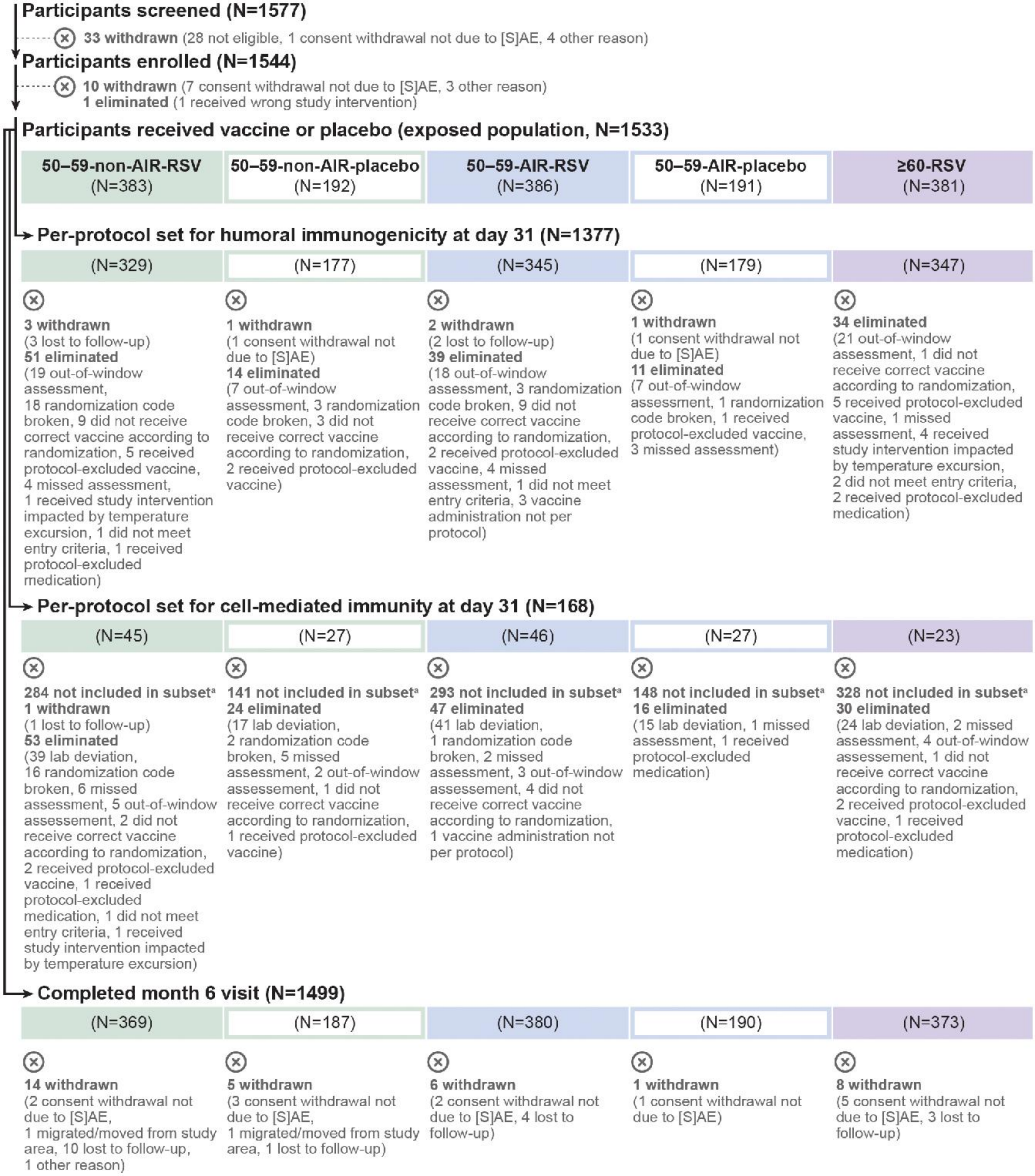
Flow of participants. For eliminations from the per-protocol sets for immunogenicity, multiple reasons could apply for 1 participant; all reasons are listed in the figure. Abbreviations: (S)AE, (serious) adverse event; 50–59-AIR-placebo, group of 50–59-year-old participants at increased risk for respiratory syncytial virus (RSV) disease who received placebo; 50–59-AIR-RSV, group of 50–59-year-old participants at increased risk for RSV disease who received RSV prefusion F protein–based vaccine (RSVPreF3 OA); 50–59-non-AIR-placebo, group of 50–59-year-old participants without increased risk for RSV disease who received placebo; 50–59-non-AIR-RSV, group of 50–59-year-old participants without increased risk for RSV disease who received RSVPreF3 OA; ≥60-RSV, group of ≥60-year-old participants who received RSVPreF3 OA. ^a^Participants in the cell-mediated immunity subset were recruited from a selected number of countries and centers (Supplementary Methods). In total, 339 participants were included in the exposed set of the cell-mediated immunity subset.

Between 28 October 2022 and 30 January 2023, 1155 adults aged 50–59 years and 387 adults aged 60 years and older were enrolled. Among the 50–59-year-old participants, 383 (non-AIR) and 386 (AIR) received RSVPreF3 OA and 192 (non-AIR) and 191 (AIR) received placebo; among those aged 60 years and older, 381 received RSVPreF3 OA. More than 95% of these participants in each group completed their month 6 visit, and more than 85% in each group were included in the day 31 per-protocol set for humoral immunogenicity (Figure 3).

Baseline characteristics were overall balanced between groups (Table 1). However, the mean body mass index tended to be higher in the two 50–59-AIR groups (30.9 kg/m^2^ and 31.3 kg/m^2^) than in the 50–59-non-AIR groups (28.4 kg/m^2^) and in the group aged 60 years and older (28.2 kg/m^2^), as did the proportions of current/former smokers (56.0% and 51.8% in the 50–59-AIR groups and 38.0–46.5% in the other groups). Over two-thirds of the participants in the two 50–59-AIR groups (69.2% and 71.2%) had exactly 1 condition associated with an increased risk for RSV disease, while the remaining 30.8% and 28.8% had at least 2 of these conditions. In the ≥60-RSV group, 24.7% had 1 such condition and 13.4% had at least 2 such conditions.

**Table 1.** Baseline Characteristics of the Participants (Exposed Population)

Characteristic	50–59-non-AIR-RSV (N = 383)	50–59-non-AIR-placebo (N = 192)	50–59-AIR-RSV (N = 386)	50–59-AIR-placebo (N = 191)	≥60-RSV (N = 381)
Mean (SD) age, y	54.8 (2.8)	54.7 (2.8)	55.3 (2.8)	55.6 (2.8)	69.5 (6.9)
Age category, n (%)					
50–59 y	383 (100)	192 (100)	386 (100)	191 (100)	0 (0.0)
60–69 y	0 (0.0)	0 (0.0)	0 (0.0)	0 (0.0)	202 (53.0)
70–79 y	0 (0.0)	0 (0.0)	0 (0.0)	0 (0.0)	130 (34.1)
≥80 y	0 (0.0)	0 (0.0)	0 (0.0)	0 (0.0)	49 (12.9)
Female sex, n (%)	221 (57.7)	119 (62.0)	186 (48.2)	85 (44.5)	188 (49.3)
Race, n (%)					
American Indian or Alaska Native	1 (0.3)	0 (0.0)	4 (1.0)	3 (1.6)	1 (0.3)
Asian	41 (10.7)	22 (11.5)	42 (10.9)	23 (12.0)	43 (11.3)
Black or African American	14 (3.7)	8 (4.2)	15 (3.9)	3 (1.6)	11 (2.9)
Native Hawaiian or Other Pacific Islander	0 (0.0)	0 (0.0)	0 (0.0)	2 (1.0)	1 (0.3)
White	320 (83.6)	158 (82.3)	324 (83.9)	158 (82.7)	324 (85.0)
Multiple	4 (1.0)	3 (1.6)	1 (0.3)	1 (0.5)	0 (0.0)
Unknown	3 (0.8)	1 (0.5)	0 (0.0)	1 (0.5)	1 (0.3)
Mean (SD) BMI, kg/m^2^	28.4 (5.9)	28.4 (6.7)	30.9 (6.8)	31.3 (7.3)	28.2 (6.0)
Smoking status (tobacco), n (%)					
Current smoker	66 (17.2)	36 (18.8)	83 (21.5)	49 (25.7)	44 (11.5)
Former smoker	99 (25.8)	37 (19.3)	133 (34.5)	50 (26.2)	133 (34.9)
Never smoker	217 (56.7)	119 (62.0)	170 (44.0)	92 (48.2)	204 (53.5)
Unknown	1 (0.3)	0 (0.0)	0 (0.0)	0 (0.0)	0 (0.0)
Any pre-existing condition,^a^ n (%)	320 (83.6)	160 (83.3)	386 (100)	191 (100)	358 (94.0)
Condition of interest,^b^ n (%)					
1 condition of interest^b^	0 (0.0)	0 (0.0)	267 (69.2)	136 (71.2)	94 (24.7)
≥2 conditions of interest^b^	0 (0.0)	0 (0.0)	119 (30.8)	55 (28.8)	51 (13.4)
Chronic pulmonary disease	0 (0.0)	0 (0.0)	148 (38.3)	79 (41.4)	59 (15.5)
Chronic cardiovascular disease	0 (0.0)	0 (0.0)	125 (32.4)	58 (30.4)	58 (15.2)
Diabetes mellitus	0 (0.0)	0 (0.0)	188 (48.7)	92 (48.2)	67 (17.6)
Chronic liver or renal disease	0 (0.0)	0 (0.0)	57 (14.8)	23 (12.0)	17 (4.5)

Abbreviations: BMI, body mass index; N, number of participants in the exposed population; SD, standard deviation; n (%), number (percentage) of participants in the indicated category; y, years; 50–59-AIR-placebo, group of 50–59-year-old participants at increased risk for respiratory syncytial virus (RSV) disease who received placebo; 50–59-AIR-RSV, group of 50–59-year-old participants at increased risk for RSV disease who received RSV prefusion F protein–based vaccine (RSVPreF3 OA); 50–59-non-AIR-placebo, group of 50–59-year-old participants without increased risk for RSV disease who received placebo; 50–59-non-AIR-RSV, group of 50–59-year-old participants without increased risk for RSV disease who received RSVPreF3 OA; ≥60-RSV, group of ≥60-year-old participants who received RSVPreF3 OA.

^a^Any pre-existing chronic condition based on the participant's medical history.

^b^Conditions of interest refer to the predefined conditions that are known to increase the risk for RSV disease (chronic pulmonary disease, chronic cardiovascular disease, diabetes mellitus types 1 and 2, chronic renal disease, and chronic liver disease).

### Immunogenicity

Both in adults 50–59 years old without an increased risk for RSV disease and in those with an increased risk for RSV disease, the immune response induced by RSVPreF3 OA was noninferior to that in those aged 60 years and older in terms of RSV-A and RSV-B neutralization titers 1 month post-vaccination. All 4 co-primary objectives were met: the ULs of the CIs around the adjusted GMT ratios were below 1.5 and the ULs of the CIs around the sero-response rate differences were below 10% for both RSV-A and RSV-B neutralization titers when comparing ≥60-RSV with 50–59-non-AIR-RSV and with 50–59-AIR-RSV groups (Table 2).

**Table 2.** Noninferiority of the RSVPreF3 OA Immune Response at 1 Month Post-vaccination in Adults 50–59 Years Old Without or With an Increased Risk for RSV Disease Versus Adults ≥60 Years Old (Per-Protocol Set for Humoral Immunogenicity)

Comparison Endpoint	Adjusted GMT Ratio (CI^a^) (≥60 Group Over 50–59 Group)	Sero-response Rate Difference (CI^a^) (≥60 Group Minus 50–59 Group)	Success Criterion Met^b^
≥60-RSV vs 50–59-non-AIR-RSV			
RSV-A neutralization titer	.95 (.83, 1.09)	−2.41 (−8.30, 3.50)	Yes
RSV-B neutralization titer	.89 (.77, 1.03)	−3.73 (−11.09, 3.68)	Yes
≥60-RSV vs 50–59-AIR-RSV			
RSV-A neutralization titer	.83 (.73, .95)	−6.47 (−12.05, −0.94)	Yes
RSV-B neutralization titer	.80 (.71, .91)	−7.15 (−13.34, −0.94)	Yes

Sero-response rate was defined as the percentage of participants with a ≥4-fold increase in neutralization titers from pre- to 1 month post-vaccination.

Abbreviations: CI, confidence interval; GMT, geometric mean titer; 50–59-AIR-RSV, group of 50–59-year-old participants at increased risk for respiratory syncytial virus (RSV) disease who received RSV prefusion F protein–based vaccine (RSVPreF3 OA); 50–59-non-AIR-RSV, group of 50–59-year-old participants without increased risk for RSV disease who received RSVPreF3 OA; ≥60-RSV, group of ≥60-year-old participants who received RSVPreF3 OA.

^a^95% CI (alpha of .025) for all comparisons except for ≥60-RSV vs 50–59-non-AIR-RSV for RSV-B neutralization titers, which used a 97.5% CI (alpha of .0125); alpha determined following the graphical testing procedure explained in the Supplementary Methods.

^b^Criterion was met if the upper limit of the CI around the adjusted GMT ratio was ≤1.5 and the upper limit of the CI around the sero-response rate difference was ≤10%.

**Figure 4.**
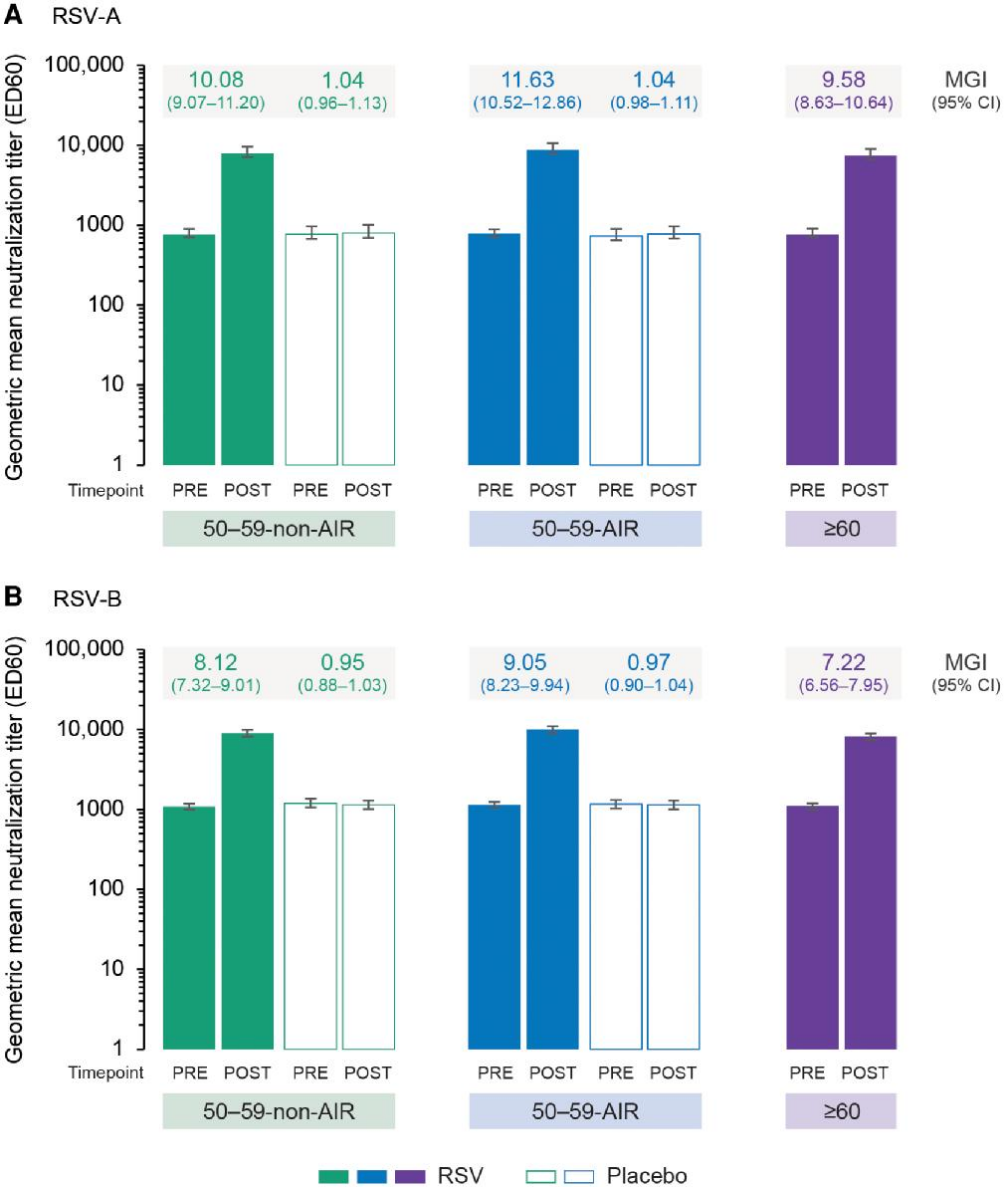
RSV-A (*A*) and RSV-B (*B*) geometric mean neutralization titers and mean geometric increases (per-protocol set for humoral immunogenicity). Confidence intervals (CIs) are depicted as error bars. Abbreviations: ED60, estimated dilution 60; MGI, mean geometric increase (ie, geometric mean of the within-participant ratios of the post-vaccination over the pre-vaccination neutralization titers); Placebo, received placebo; POST, 1 month after vaccination; PRE, before vaccination; RSV, respiratory syncytial virus; 50–59-AIR, group of 50–59-year-old participants at increased risk for RSV disease; 50–59-non-AIR, group of 50–59-year-old participants without increased risk for RSV disease; ≥60, group of ≥60-year-old participants.

The RSV-A and RSV-B neutralization titers increased markedly from pre- to 1 month post-vaccination in the different groups of RSVPreF3 OA–vaccinated participants, with mean geometric increases ranging from 9.58 to 11.63 for RSV-A and 7.22 to 9.05 for RSV-B (Figure 4), and sero-response rates ranging from 80.4% to 86.9% for RSV-A and 74.5% to 81.6% for RSV-B (Supplementary Table 1). No increases in RSV-A and RSV-B neutralization titers were seen in the placebo groups. Observed post-vaccination GMTs for RSV-A and RSV-B were numerically highest in the 50–59-AIR-RSV group (8821.9 and 9967.3 ED60, respectively), followed by the 50–59-non-AIR-RSV group (7925.4 and 8971.9 ED60) and the ≥60-RSV group (7461.9 and 8144.5 ED60), but 95% CIs generally overlapped (Figure 4).

**Figure 5.**
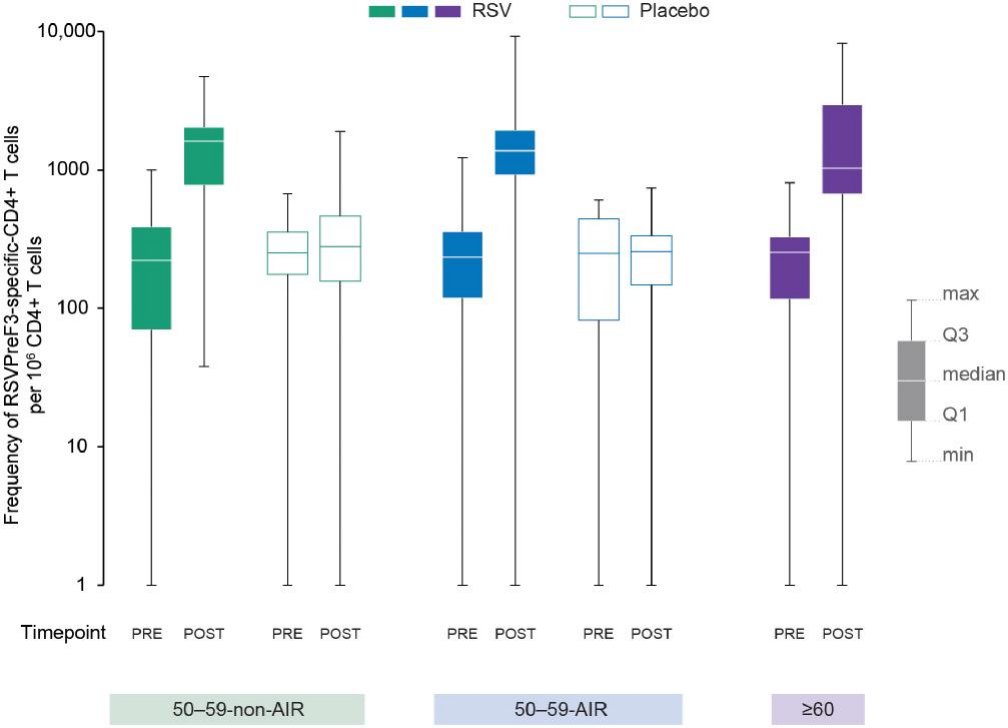
Frequency of RSVPreF3-specific-CD4+ T cells expressing ≥2 markers including ≥1 cytokine among CD40L, 4-1BB, IL-2, TNF-α, IFN-γ, IL-13, and IL-17 (per-protocol set for cell-mediated immunity). Abbreviations: CD40L, CD40 ligand; IFN-γ, interferon-γ; IL, interleukin; max, maximum; min, minimum; Placebo, received placebo; POST, 1 month after vaccination; PRE, before vaccination; Q1, first quartile; Q3, third quartile; RSV, received respiratory syncytial virus (RSV) prefusion F protein–based vaccine (RSVPreF3 OA); RSVPreF3, respiratory syncytial virus prefusion F protein; TNF-α, tumor necrosis factor-α; 50–59-non-AIR, group of 50–59-year-old participants without increased risk for RSV disease; 50–59-AIR, group of 50–59-year-old participants at increased risk for RSV disease; ≥60, group of ≥60-year-old participants.

The frequencies of RSVPreF3-specific CD4+ T cells expressing 2 or more activation markers including 1 or more cytokine among CD40L, 4-1BB, IL-2, TNF-α, IFN-γ, IL-13, and IL-17 increased substantially from pre- to 1 month post-vaccination in all groups of RSVPreF3 OA–vaccinated participants, reaching similar post-vaccination frequencies in the 50–59-non-AIR-RSV (median, 1616.0/10^6^ CD4+ T cells), 50–59-AIR-RSV (1379.0/10^6^ CD4+ T cells), and ≥60-RSV (1033.0/10^6^ CD4+ T cells) groups (Figure 5). No increase in CD8+ T-cell frequencies was detected in any of the groups.

### Reactogenicity and Safety

**Figure 6.**
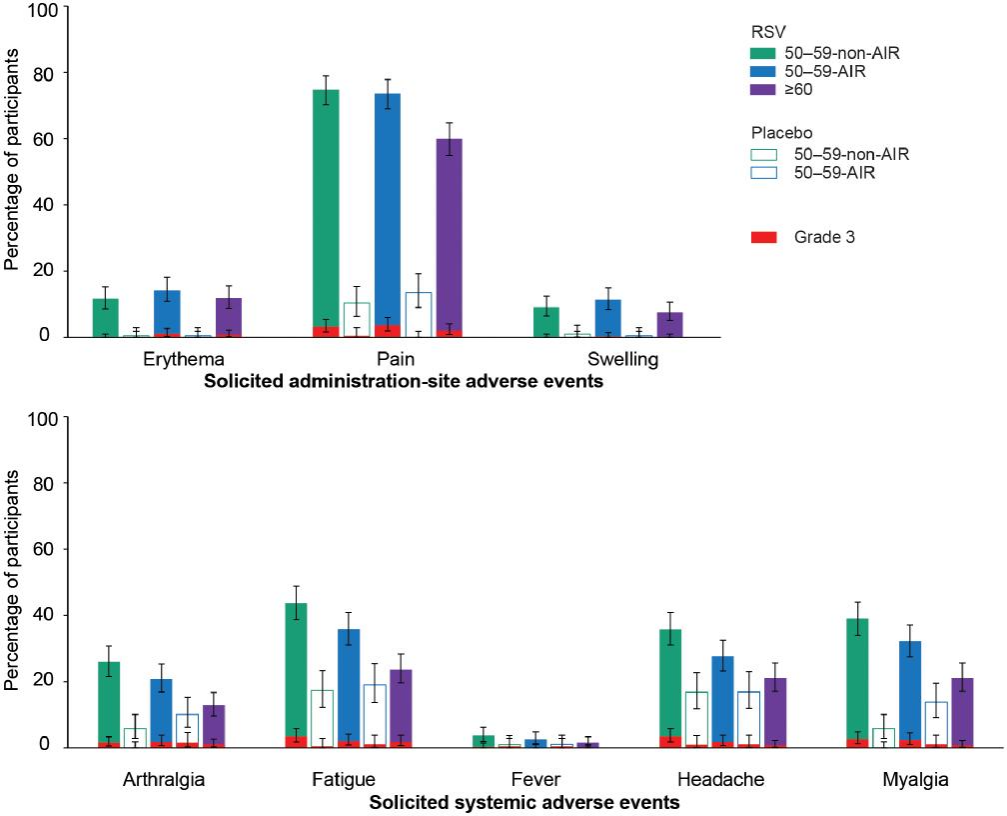
Solicited adverse events within 4 days after RSVPreF3 OA or placebo administration (exposed population). Confidence intervals are depicted as error bars. Fever was defined as a temperature ≥38.0°C. Grade 3 adverse events were defined as administration-site erythema or swelling with a diameter >100 mm, fever with a temperature >39.0°C, and administration-site pain, headache, fatigue, myalgia, and arthralgia that prevented normal activity. Abbreviations: Placebo, received placebo; RSV, received respiratory syncytial virus (RSV) prefusion F protein–based vaccine (RSVPreF3 OA); 50–59-non-AIR, group of 50–59-year-old participants without increased risk for RSV disease; 50–59-AIR, group of 50–59-year-old participants at increased risk for RSV disease; ≥60, group of ≥60-year-old participants.

In all groups, the most common solicited AEs starting within 4 days post–RSVPreF3 OA vaccination were administration-site pain, fatigue, myalgia, and headache. These events, as well as arthralgia, were reported more frequently among RSVPreF3 OA–vaccinated 50–59-year-olds than in those aged 60 years and older (Figure 6, Supplementary Table 2). The median duration of each solicited AE was similar across all vaccine and placebo groups and was 4 days or less for administration-site AEs and 3 days or less for systemic AEs. The reporting rates of solicited AEs with grade 3 intensity were low (≤3.7% for any event in any group) and similar between groups. Grade 3 fever (temperature >39.0°C) was reported for 0.5% or less of participants across groups (Figure 6, Supplementary Table 2).

Unsolicited AEs with onset within 30 days post-vaccination were reported for 10.5–14.5% of 50–59-year-olds across the 4 groups and for 16.3% of those aged 60 years and older (Table 3). Within this period, 1 case of new-onset atrial fibrillation (nonserious AE) was reported in the ≥60-RSV group, which was not considered as being related to vaccination by the investigator.

**Table 3.** Unsolicited Adverse Events, Serious Adverse Events, and Potential Immune-Mediated Diseases After RSVPreF3 OA or Placebo Administration (Exposed Population)

Adverse Event	50–59-non-AIR-RSV (N = 383)		50–59-non-AIR-placebo (N = 192)		50–59-AIR-RSV (N = 386)		50–59-AIR-placebo (N = 191)		≥60-RSV (N = 381)	
	n	% (95% CI)	n	% (95% CI)	n	% (95% CI)	n	% (95% CI)	n	% (95% CI)
Unsolicited AEs within 30 days										
Any	50	13.1 (9.8, 16.8)	26	13.5 (9.0, 19.2)	56	14.5 (11.1, 18.4)	20	10.5 (6.5, 15.7)	62	16.3 (12.7, 20.4)
Grade 3	4	1.0 (.3, 2.7)	0	0.0 (.0, 1.9)	4	1.0 (.3, 2.6)	4	2.1 (.6, 5.3)	2	.5 (.1, 1.9)
Related	14	3.7 (2.0, 6.1)	5	2.6 (.9, 6.0)	12	3.1 (1.6, 5.4)	3	1.6 (.3, 4.5)	12	3.1 (1.6, 5.4)
Grade 3 related	0	0.0 (.0, 1.0)	0	0.0 (.0, 1.9)	0	0.0 (.0, 1.0)	0	0.0 (.0, 1.9)	1	.3 (.0, 1.5)
Medically attended	12	3.1 (1.6, 5.4)	10	5.2 (2.5, 9.4)	24	6.2 (4.0, 9.1)	13	6.8 (3.7, 11.4)	27	7.1 (4.7, 10.1)
SAEs and pIMDs within 6 months										
Any SAE	2	.5 (.1, 1.9)	4	2.1 (.6, 5.2)	14	3.6 (2.0, 6.0)	4	2.1 (.6, 5.3)	9	2.4 (1.1, 4.4)
Any pIMD^a^	0	0.0 (.0, 1.0)	0	0.0 (.0, 1.9)	4	1.0 (.3, 2.6)	1	.5 (.0, 2.9)	3	.8 (.2, 2.3)
Related or fatal SAEs and related pIMDs until data lock point										
Related SAE	0	0.0 (.0, 1.0)	0	0.0 (.0, 1.9)	0	0.0 (.0, 1.0)	0	0.0 (.0, 1.9)	1	0.3 (.0, 1.5)
Fatal SAE	0	0.0 (.0, 1.0)	0	0.0 (.0, 1.9)	0	0.0 (.0, 1.0)	0	0.0 (.0, 1.9)	0	0.0 (.0, 1.0)
Related pIMD	0	0.0 (.0, 1.0)	0	0.0 (.0, 1.9)	0	0.0 (.0, 1.0)	0	0.0 (.0, 1.9)	1	0.3 (.0, 1.5)

A grade 3 adverse event (AE) is an AE that prevents normal activity.

Abbreviations: CI, confidence interval; N, number of participants in the exposed population; n (%), number (percentage) of participants in the indicated category; pIMD, potential immune-mediated disease; SAE, serious adverse event; 50–59-AIR-RSV, group of 50–59-year-old participants at increased risk for respiratory syncytial virus (RSV) disease who received RSV prefusion F protein–based vaccine (RSVPreF3 OA); 50–59-non-AIR-RSV, group of 50–59-year-old participants without increased risk for RSV disease who received RSVPreF3 OA; ≥60-RSV, group of ≥60-year-old participants who received RSVPreF3 OA.

^a^Reported pIMDs were new-onset pericarditis, new-onset spondylitis, worsening of pre-existing gouty arthritis, and worsening of pre-existing gout in the 50–59-AIR-RSV group, pericarditis in the 50–59-AIR-placebo group, and new-onset cold-type hemolytic anemia, new-onset polymyalgia rheumatica, and worsening of pre-existing psoriasis in the ≥60-RSV group.

Up to 6 months post-vaccination, 0.5–3.6% of 50–59-year-old participants and 2.4% of participants aged 60 years and older reported SAEs, and 0.0–1.0% of 50–59-year-olds and 0.8% of those aged 60 years and older reported pIMDs (Table 3). One case of new-onset atrial fibrillation, which the investigator judged as not being vaccination-related, was reported as an SAE in the ≥60-RSV group. Up to the data lock point for this analysis, 1 SAE (also reported as pIMD), a case of cold-type hemolytic anemia in the ≥60-RSV group, was considered by the investigator as being related to RSVPreF3 OA vaccination. This case started 52 days after vaccination and was ongoing at the data lock point. The participant had multiple hematology lab results showing anemia, raised bilirubin levels, and raised lactate dehydrogenase levels in past medical checkups before receiving RSVPreF3 OA.

No deaths were reported up to the data lock point.

## DISCUSSION

This study demonstrated that the humoral immune response induced by RSVPreF3 OA in adults aged 50–59 years was noninferior to that in adults aged 60 years and older. Noninferiority was demonstrated for 50–59-year-olds without and for those with chronic conditions that increase the risk for RSV disease, both for RSV-A and RSV-B neutralization titers. Given that RSVPreF3 OA efficacy was demonstrated in adults aged 60 years and older [7–9], these results suggest that RSVPreF3 OA may also be efficacious in 50–59-year-olds, both in those without and those with underlying conditions that increase the risk for RSV disease.

A robust humoral immune response was seen in all 3 RSVPreF3 OA-vaccinated groups. Interestingly, post-vaccination RSV-A and RSV-B neutralization GMTs were not lower (rather, numerically higher) in the AIR than in the non-AIR subcohort, despite the trend for a greater body mass index and proportion of smokers in this subcohort, 2 factors that have been associated with lower immune responses to some vaccines in several studies [16]. Similarly, among those aged 60 years and older in the phase 3 efficacy study, a trend for higher RSV neutralization titers was observed in participants with underlying medical conditions [9], and the vaccine efficacy against RSV-LRTD tended to be higher in this population (94.6% over 1 RSV season) compared with the overall population (82.6%) [7, 9].

We also observed numerically higher neutralization titers in adults aged 50–59 years than in those aged 60 years and older. A trend for lower RSV neutralization titers in older age groups was seen previously after RSVPreF3 OA immunization [17]. However, the clinical relevance of these observations is not known as our study was not designed to detect between-group differences in immune responses, and 95% CIs between groups mostly overlapped.

Vaccination with RSVPreF3 OA also induced a robust CD4+ T-cell response in all groups. This increase in CD4+ T-cell frequencies is important as T-cell immunity is thought to play a role in protecting against RSV disease and controlling disease severity [18–22]. Consistent with previous studies in individuals aged 60 years and older [17, 23], RSVPreF3 OA did not induce an increase in CD8+ T cells in 50–59-year-olds.

Several solicited AEs were reported at higher rates in 50–59-year-olds than in those aged 60 years and older. Higher vaccine reactogenicity in younger versus older adults was not unexpected as it has been observed with other vaccines [24–26]. However, symptoms were transient, and incidences of grade 3 reactions were similarly low among 50–59-year-olds and those aged 60 years and older. The rates of unsolicited AEs, SAEs, and pIMDs were balanced between study groups. Hence, the overall safety profile in adults aged 50–59 years was consistent with the known safety profile in those aged 60 years and older.

Respiratory syncytial virus poses a significant burden in 50–59-year-olds, with increased hospitalization rates and a greater use of medical resources among those with underlying medical conditions [1–4, 27]. Respiratory syncytial virus can also compromise the quality of life in this population, leading to an impairment in daily functioning [14, 15]. Considering that a substantial proportion of adults aged 50–59 years are living with conditions that increase the risk for RSV disease, such as chronic obstructive pulmonary disease or diabetes [28–30], an effective vaccine in this population could help decrease the total RSV burden.

A strength of our study was the inclusion of several internal control groups: a non-AIR subcohort of 50–59-year-olds, placebo groups for both the 50–59-AIR and 50–59-non-AIR subcohorts, and a cohort of participants aged 60 years and older that was similar to the population in which efficacy was demonstrated. Including these in the same study allowed us to control for possible differences in seasonality or regionality in RSV epidemiology. Another strength was the heterogenous study population from different geographic areas, providing good external validity.

Our study has some limitations. There was no surveillance for RSV-LRTD, and as there is no established correlate of protection against RSV, we do not have direct evidence for efficacy in 50–59-year-olds. However, the high efficacy previously demonstrated in those aged 60 years and older, including in those with chronic conditions associated with an increased risk for RSV disease [7–9], together with the comparable immune response observed in this study are highly suggestive of the vaccine being efficacious in adults aged 50–59 years with or without the predefined chronic conditions. Additional limitations are the inclusion of participants with stable chronic conditions only and the exclusion of adults with immunocompromising conditions, who also have an increased risk for severe RSV outcomes [5, 6]. Also, some conditions that may increase the risk for RSV disease (eg, neurological or neuromuscular disorders [5]) were not specifically selected in the AIR subcohort or excluded from the non-AIR subcohort. Finally, the primary objective of our study was to demonstrate noninferiority comparing the 50–59-RSV groups with the ≥60-RSV group; the study was not designed to show differences in immune responses between all of the study groups. Neither was it powered to identify rare AEs following vaccination.

In summary, this study demonstrated that RSVPreF3 OA was at least as immunogenic in adults aged 50–59 years (with or without increased risk for RSV disease) as in those aged 60 years and older, and showed that RSVPreF3 OA had an acceptable safety profile in 50–59-year-olds consistent with that in those aged 60 years and older. Together with the previously demonstrated efficacy in the older age group, these results suggest that RSVPreF3 OA could provide a substantial clinical benefit to 50–59-year-olds and help address the unmet medical need in this population.

## Supplementary Data

Supplementary materials are available at *Clinical Infectious Diseases* online. Consisting of data provided by the authors to benefit the reader, the posted materials are not copyedited and are the sole responsibility of the authors, so questions or comments should be addressed to the corresponding author.

## Supplementary Material

ciae364_Supplementary_Data

## Notes

***Author contributions*.** H. A. H., M.-P. D., D. D., J. H., and C. V. were involved in the conception or design of the study/project. M. F., T. F. S., S. A. N., J. R.-G., M. M., C. Z., B. S., N. T., D. O. M., J. G., C. V.-P., H. M., M. d. H., and B. S. participated in the acquisition or generation of the data. S. D., M.-P. D., J. H., C. V., and V. H. were involved in the data analysis. T. F. S., H. A. H., B. S., S. D., D. D., J. H., C. V., and V. H. were involved in the interpretation of the data.

***Acknowledgments***. The authors are grateful to all participants, study team members, investigators, and study site staff for their contributions to the study. They are grateful to Catherine Gérard and the staff at GSK Vaccines Clinical Laboratory and Assay Portfolio laboratories for their technical assistance, to Mathilde Laffitte for contributing to the data analysis, to Mathieu Peeters for support in study concept and design, and to Loïc François (Akkodis Belgium, c/o GSK) for his involvement in the data analysis and in the interpretation of the data. They also thank Akkodis Belgium (c/o GSK) for medical writing (by Natalie Denef), design, and publication coordination support.

***RSV OA=ADJ-018 Study Group members***. Khalid S. Abd-Elaziz, Mark S. Adams, Agnieszka Barts, Kevin Cannon, Matthew Davis, Sonia de las Fuentes Galán, Marta de los Ríos Rodríguez, Maria Cristina De Salvo, Lauren DeGregoria, Víctor del Campo Pérez, Torsten Drescher, Rebecca Dunsmoor-Su, Peter Dzongowski, Jose Ma Echave-Sustaeta, Tamara Julia Eckermann, Ashley E. Fuller, Jaume Garí Parera, Jean Sebastien Gauthier, Steven Geller, Wayne Ghesquiere, Antonio Gonzalez, Patricia González Cediel, Anton Grasch, Laura L. Helman, Susan Hernandez, María Herranz Urbasos, Nicolas Itcovici, Terry Klein, Jorge Labrador Gómez, Antonio Lalueza Blanco, Ryan Leblanc, Matthias Luttermann, Kristen Marks, Cristina Masuet-Aumatell, Leonie Möckesch, Tamara Michelle Moreno Silva, Silvia Narejos Perez, Robert J. Noveck, Jérôme C. Oude Nijhuis, Jean-Sebastien Paquette, Bonavuth Pek, Georg Plassmann, Robert Pritt, Mireia Puig Palma, Claudio Rocha-Calderon, Paule Royer, David Shu, Ying Tung Sia, Angelika Sieber, Todd Simmons, Leslie Sinclair, William B. Smith, Joseph Soufer, Ana Suarez Simón, Genoveva Vilardell Rifa, María Teresa Vilella Moreno, Ulrich Weber, Alba María Yañez de la Higuera, and Pedro Ylisastigui.

***Data-sharing statement***. Anonymized data can be requested from www.clinicalstudydatarequest.com.

***Trademarks***. Arexvy and AS01_E_ are trademarks licensed to or owned by GSK.

***Financial support*.** This work was supported by GlaxoSmithKline Biologicals SA, which funded this trial, was involved in all stages of trial conduct, and took charge of all costs associated with the development and publication of this manuscript.
