# Supplementary material for: Noninferior Immunogenicity and Consistent Safety of Respiratory Syncytial Virus Prefusion F Protein Vaccine in Adults 50–59 Years Compared to ≥60 Years of Age
Source: Clin Infect Dis. 2024 Aug 5;79(4):1074–84. doi: 10.1093/cid/ciae364 (PMC11478578; doi:10.1093/cid/ciae364)
Supplement: ciae364_Supplementary_Data [file ciae364_supplementary_data.docx]

**Supplementary material**

**Supplementary methods**

***Inclusion criteria***

*General criteria*

- Participants who, in the opinion of the investigator, could and would comply with the requirements of the protocol (e.g., completion of the diary cards, attending study site visits, ability to access and use a phone or other electronic communications).
  - Note: In case of physical incapacity that would preclude self-completion of the diary cards, either site staff could assist the participant (for activities performed during site visits) or the participant could assign a caregiver to assist with this activity (for activities performed at home). However, at no time could the site staff or caregiver evaluate the participant’s health status while completing diaries or make decisions on behalf of the participant.
- Written or witnessed informed consent obtained from the participant prior to performance of any study-specific procedure.

*Specific criteria for 50–59-year-old participants*

- A male or female participant 50–59 years of age at the time of the study intervention administration.
- Female participants of non-childbearing potential could be enrolled in the study. Non-childbearing potential was defined as hysterectomy, bilateral oophorectomy, bilateral salpingectomy, or post-menopause.
- Female participants of childbearing potential could be enrolled in the study if the participant:
  - had practiced adequate contraception from 1 month prior to study intervention administration until study end, and
  - had a negative pregnancy test on the day of study intervention administration.

*Specific criteria for 50–59-year-old participants not at increased risk for respiratory syncytial virus (RSV) disease (non-AIR)*

- Healthy participants as established by medical history and clinical examination before entering the study.
- Participants with chronic stable medical conditions with or without specific treatment, such as hypertension, hypercholesterolemia, or hypothyroidism, and who were not at increased risk for RSV-associated lower respiratory tract disease (RSV-LRTD) were allowed to participate in this study if considered by the investigator as medically stable (no changes in treatment or disease severity in the past 3 months).

*Specific criteria for 50–59-year-old participants at increased risk for RSV disease (AIR)*

- Participants had to be diagnosed with at least one of the following medical conditions and have a stable condition (no changes in the treatment or disease severity in the past 3 months):
  - Chronic pulmonary disease resulting in activity restricting symptoms or use of long-term medication:
    - Chronic obstructive pulmonary disease (COPD): Global Initiative for COPD grade 2–4
    - Asthma: patient on regular medication (excluding exercise asthma)
    - Cystic fibrosis
    - Other chronic respiratory diseases: lung fibrosis, restrictive lung disease, interstitial lung disease, emphysema, or bronchiectasis
  - Chronic cardiovascular disease:
    - Chronic heart failure: a minimum of class II symptoms according to New York Heart Association classification of heart failure
    - Pre-existing coronary artery disease (CAD not otherwise specified): physician diagnosis of CAD based on electrocardiogram, exercise stress test, nuclear stress test, cardiac computed tomography scan, or cardiac angiogram (more than the presence of hypercholesterolemia)
    - Cardiac arrhythmia: patient on treatment for cardiac arrhythmia
  - Diabetes mellitus: types 1 and 2
  - Other diseases increasing the risk for RSV-LRTD:
    - Chronic kidney disease: G2–G3 disease (glomerular filtration rate between 30 and 90 ml/min/1.73 m^2^)
    - Chronic liver disease

*Specific criteria for ≥60-year-old participants*

- A male or female participant ≥60 years of age at the time of the study intervention administration.
- Participants with chronic stable medical conditions with or without specific treatment, such as diabetes, hypertension, or cardiac disease were allowed to participate in this study if considered by the investigator as medically stable (no changes in the treatment or disease severity in the past 3 months).
- Participants living in the general community or in an assisted-living facility that provides minimal assistance, such that the participant is primarily responsible for self-care and activities of daily living.

***Exclusion criteria***

*Medical conditions*

- Any confirmed or suspected immunosuppressive or immunodeficient condition resulting from disease (e.g., current malignancy, human immunodeficiency virus) or immunosuppressive/cytotoxic therapy (e.g., medication used during cancer chemotherapy, organ transplantation, or to treat autoimmune disorders), based on medical history and physical examination (no laboratory testing required).
- History of any reaction or hypersensitivity likely to be exacerbated by any component of the study intervention.
- Hypersensitivity to latex.
- Unstable chronic illness.
- Any history of dementia or any medical condition that moderately or severely impairs cognition.
  - Note: If deemed necessary for clinical evaluation, the investigator could use tools such as Mini-Mental State Exam, Mini-Cog, or Montreal Cognitive Assessment to determine cognition levels of the participant.
- Recurrent or uncontrolled neurological disorders or seizures. Participants with medically controlled active or chronic neurological diseases could be enrolled in the study as per investigator assessment, provided that their condition would allow them to comply with the requirements of the protocol (e.g., completion of the diary cards, attending study site visits). Study participants could decide to assign a caregiver to help them complete the study procedures.
- Significant underlying illness that in the opinion of the investigator would be expected to prevent completion of the study (e.g., life-threatening disease).
- Any medical condition that in the judgment of the investigator would make intramuscular injection unsafe.

*Prior and concomitant therapy*

- Use of any investigational or non-registered product (drug, vaccine, or medical device) other than the study intervention during the period beginning 30 days before the dose of study intervention (day -29 to day 1), or planned use during the study period (up to month 12, visit 4).
- Planned or actual administration of a vaccine not foreseen by the study protocol in the period starting 30 days before and ending 30 days after the dose of study intervention administration, with the exception of inactivated and subunit influenza vaccines or COVID-19 vaccines (fully licensed or with emergency use authorization) which could be administered up to 14 days before or from 14 days after the study intervention administration.
  - Note: In case an emergency mass vaccination for an unforeseen public health threat (e.g., a pandemic) was recommended and/or organized by the public health authorities, outside the routine immunization program, the period described above could be reduced if necessary for that vaccine provided it was used according to the local governmental recommendations and that the Sponsor was notified accordingly.
- Previous vaccination with an RSV vaccine, including investigational RSV vaccines.
- Chronic administration of immune-modifying drugs (defined as more than 14 consecutive days in total) and/or administration of long-acting immune-modifying treatments or planned administration at any time up to the end of the study.
  - Up to 3 months prior to the study intervention administration:
    - For corticosteroids, this meant prednisone ≥20 mg/day, or equivalent. Inhaled and topical steroids were allowed.
    - Administration of immunoglobulins and/or any blood products or plasma derivatives.
  - Up to 6 months prior to study intervention administration:
    - Long-acting immune-modifying drugs including, among others, immunotherapy (e.g., tumor necrosis factor-inhibitors), monoclonal antibodies, antitumoral medication.

*Prior and concurrent clinical study experience*

- Concurrently participating in another clinical study, at any time during the study period, in which the participant was or would be exposed to an investigational or a non-investigational vaccine/product (drug or invasive medical device).

*Other exclusions for all participants*

- History of chronic alcohol consumption and/or drug abuse as deemed by the investigator to render the potential participant unable/unlikely to provide accurate safety reports or comply with study procedures.
- Bedridden participants.
- Planned move during the study period that would prohibit participating in the study until study end.
- Participation of any study personnel or their immediate dependents, family, or household members.

*Other exclusions for 50–59-year-old participants*

- Pregnant or lactating female.
- Female planning to become pregnant or planning to discontinue contraceptive precautions.

***Enrollment rules***

Enrollment in the cohort of 50–59-year-olds was done to ensure that the AIR and non-AIR subcohorts each included approximately 50% of the 50–59-year-old participants. In the AIR subcohort, enrollment rules were implemented to ensure adequate representation of the different diseases by aiming to include approximately 25% of participants with chronic pulmonary disease, 25% with chronic cardiovascular disease, and 25% with diabetes mellitus types 1 and 2. The remaining 25% could be distributed freely across these three disease categories and could include participants with chronic renal or liver disease.

Enrollment in the cohort of ≥60-year-olds was done to ensure adequate representation of the different age categories (approximately 40% in the 60–69 years, 30% in the 70–79 years, and 10% in the ≥80 years category, with the remaining 20% distributed freely across these three age categories).

***Vaccine composition, randomization, and blinding***

Each 0.5-ml dose of reconstituted RSV prefusion F protein-based vaccine (RSVPreF3 OA) contained 120 μg of RSVPreF3 antigen and AS01_E_, an adjuvant system containing 25 μg of 3-O-desacyl-4′-monophosphoryl lipid A, 25 μg of QS21 (*Quillaja saponaria* Molina, fraction 21, licensed by GSK from Antigenics LLC, a wholly owned subsidiary of Agenus Inc., a Delaware, USA corporation), and liposome. Placebo was NaCl solution. Both vaccine and placebo were administered intramuscularly in the deltoid of the (preferably) non-dominant arm.

Participants were randomized using an automated internet-based system. The randomization algorithm used a stratification by AIR/non-AIR status and cell-mediated immunity (CMI) subset (participant included in the CMI subset or not) and a minimization procedure accounting for the study and center within each stratum. Minimization factors had equal weight in the minimization algorithm.

Data from the cohort of ≥60-year-olds were collected in an open-label manner. Until the time of the analysis of the primary endpoints, data from the cohort of 50–59-year-olds were collected in an observer-blind manner. The participants and site personnel involved in the clinical evaluation of the participants were blinded to the study intervention. Therefore, RSVPreF3 OA and placebo were prepared and administered by site personnel that was not involved in the evaluation of study endpoints. The laboratory in charge of sample testing was blinded to the study intervention. After the analysis of the primary endpoints, the study was considered single-blind. The participants in the cohort of 50–59-year-olds remained blinded until study end. Investigators also remained blinded overall, but some could become unblinded to certain participants through summary results in the study report (which they received after analysis of the primary endpoints, albeit without individual data listings and participant treatment assignments).

***CMI subset***

The CMI subset was to include approximately 350 participants (100 each in 50–59-non-AIR-RSV and 50–59-AIR-RSV, 50 each in 50–59-non-AIR-placebo and 50–59-AIR-placebo, and 50 in ≥60-RSV). Participants in the CMI subset were recruited from a selected number of countries and centers. In the selected centers, the investigator allocated the first participants in each cohort/subcohort to the CMI subset until the allocated target was reached.

***CMI laboratory assay***

For the intracellular cytokine staining, thawed peripheral blood mononuclear cells were stimulated in vitro in the presence of anti-CD28 and anti-CD49d antibodies either with pools of 15-mer peptides overlapping by 11 amino acids and spanning the sequence of the RSVPreF3 protein, or with medium. After 2 hours of incubation at 37°C, Brefeldin A was added to inhibit cytokine secretion during an additional overnight incubation at 37°C. Cells were subsequently harvested, stained for surface markers (CD4+ and CD8+) and then fixed. Fixed cells were then permeabilized and stained with labeled antibodies specific for the following immune markers: CD3+ (to phenotype T cells), CD40 ligand (CD154), 4-1BB (CD137), Interleukin (IL)-2, Tumor necrosis factor-α, Interferon-γ, IL-13, and IL-17. After staining for these markers, the cellular samples were analyzed by flow cytometry to determine the frequency of CD4+ and CD8+ T cells expressing the marker(s) of interest per million of CD4+ and/or CD8+ T cells.

***Sample size***

The planned sample size was approximately 1520 participants: 380 participants each in the 50–59-non-AIR-RSV and 50–59-AIR-RSV groups, 190 each in the 50–59-non-AIR-placebo and 50–59-AIR-placebo groups, and 380 in the ≥60-RSV group.

The sample size in the groups receiving RSVPreF3 OA was driven by the statistical power to demonstrate the co-primary non-inferiority objectives. Assuming that 10% of enrolled participants would be eliminated from the per-protocol set, the planned sample size would result in 342 participants evaluable for the primary objectives in each group receiving RSVPreF3 OA. With 342 participants per group, the overall power to demonstrate the four co-primary objectives following the graphical testing procedure described in the ‘Statistical analysis’ section below would be 82.7%.

| **Objective** | **Power** |
| --- | --- |
| Non-inferiority in the 50–59-non-AIR-RSV group compared to the ≥60-RSV group for RSV-A | >99% |
| Non-inferiority in the 50–59-non-AIR-RSV group compared to the ≥60-RSV group for RSV-B | >99% |
| Non-inferiority in the 50–59-AIR-RSV group compared to the ≥60-RSV group for RSV-A | 93.6% |
| Non-inferiority in the 50–59-AIR-RSV group compared to the ≥60-RSV group for RSV-B | 82.8% |
| **Overall** | **82.7%** |

***Statistical analyses***

The two-sided 95% and 97.5% confidence intervals (CIs) for the adjusted geometric mean titer (GMT) ratios (≥60-RSV over 50–59-non-AIR-RSV and ≥60-RSV over 50–59-AIR-RSV) were derived from an analysis of covariance model on log_10_-transformed neutralization titers for RSV-A and RSV-B. The model included the group and the baseline log_10_-transformed titer as covariates.

The two-sided 95% and 97.5% CIs for the group seroresponse rate differences (≥60-RSV minus 50–59-non-AIR-RSV and ≥60-RSV minus 50–59-AIR-RSV) were derived using the method of Miettinen and Nurminen [1].

Missing data were not replaced.

The following graphical testing procedure was applied to control the global type I error at 2.5% (one-sided) [2].


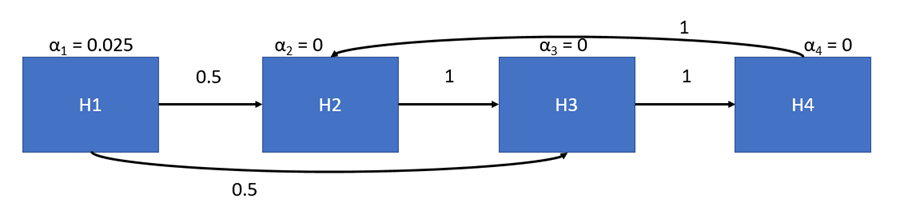


H1, null hypothesis 1: adjusted GMT ratio (≥60-RSV/50–59-non-AIR-RSV) is >1.5 or seroresponse rate difference (≥60-RSV minus 50–59-non-AIR-RSV) is >10% for RSV-A at 1 month post-vaccination; H2, null hypothesis 2: same as H1 but for RSV-B; H3, null hypothesis 3: adjusted GMT ratio (≥60-RSV/50–59-AIR-RSV) is >1.5 or seroresponse rate difference (≥60-RSV minus 50–59-AIR-RSV) is >10% for RSV-A at 1 month post-vaccination; H4, null hypothesis 4, same as H3 but for RSV-B. Null hypotheses had to be rejected in favor of the alternative hypothesis that the GMT ratios were ≤1.5 and seroresponse rate differences were ≤10%.

The initial allocation of α among the null hypotheses was (0.025, 0, 0, 0), and the propagation rules were specified by the transition matrix:


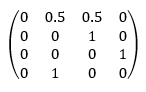


The hypothesis testing started with H1 at a significance level α1 of 2.5%. If H1 was rejected, the sequence would move to H2 and H3 testing and propagate the significance level as 0.5*α1 to both, updating α2 and α3 to 1.25%. If H2 was rejected, the sequence would then move to H3 testing at a significance level of 1*α2 (1.25%). H3 was subsequently tested with an updated alpha level α3 of 2.5% (i.e., 0.5*α1 coming from H1 and 1*α2 coming from H2). If H3 was rejected, α3 would then be propagated to H4 for testing at the updated level α4 of 2.5% (1*α3).

To compute GMTs, seroresponse rates, and mean geometric increases, RSV-A and RSV-B neutralization titers below the lower limits of quantification (LLOQs) were replaced by half the LLOQs and titers above the upper limits of quantification (ULOQs) were replaced by the ULOQs.

The RSVPreF3-specific CD4+ and CD8+ T-cell frequencies were obtained by subtracting the background frequency from the RSVPreF3 antigen-induced frequency, and by setting to 1 all values ≤0 for geometric mean calculation and graphical representation. Background frequencies were calculated as the number of CD4+ or CD8+ T cells expressing ≥2 activation markers including ≥1 cytokine over the total number of CD4+ or CD8+ T cells in the assay, after stimulation with medium only. RSVPreF3 antigen-induced frequencies were calculated as the number of CD4+ or CD8+ T cells expressing ≥2 activation markers including ≥1 cytokine over the total number of CD4+ or CD8+ T cells in the assay, after stimulation with a pool of peptides covering RSVPreF3. Frequencies were expressed as the number of cells per million of CD4+ or CD8+ T cells.

**Supplementary tables**

**Supplementary table 1. Seroresponse rates at 1 month after RSVPreF3 OA or placebo administration (per-protocol set for humoral immunogenicity)**

| **Parameter** | **50–59-non-AIR-RSV**  **N=326** | **50–59-non-AIR-placebo**  **N=175** | **50–59-AIR-RSV**  **N=343** | **50–59-AIR-placebo**  **N=176** | **≥60-RSV**  **N=342^a^** |
| --- | --- | --- | --- | --- | --- |
| **RSV-A neutralization titers** | | | | | |
| n | 270 | 5 | 298 | 1 | 275 |
| % (95% CI) | 82.8 (78.3, 86.8) | 2.9 (0.9, 6.5) | 86.9 (82.8, 90.3) | 0.6 (0.0, 3.1) | 80.4 (75.8, 84.5) |
| **RSV-B neutralization titers** | | | | | |
| n | 255 | 3 | 280 | 1 | 254 |
| % (95% CI) | 78.2 (73.3, 82.6) | 1.7 (0.4, 4.9) | 81.6 (77.1, 85.6) | 0.6 (0.0, 3.1) | 74.5 (69.5, 79.0) |

Seroresponse rate was defined as the percentage of participants with an ≥4-fold increase in neutralization titers from pre- to 1 month post-vaccination. 50–59-non-AIR-RSV/placebo, group of 50–59-year-old participants without increased risk for respiratory syncytial virus (RSV) disease who received RSV prefusion F protein-based vaccine (RSVPreF3 OA)/placebo; 50–59-AIR-RSV/placebo, group of 50–59-year-old participants at increased risk for RSV disease who received RSVPreF3 OA/placebo; ≥60-RSV, group of ≥60-year-old participants who received RSVPreF3 OA; N, number of participants with available results pre- and post-vaccination; n/%, number/percentage of participants with a seroresponse; CI, confidence interval.

^a^N=341 for RSV-B.

**Supplementary table 2. Solicited adverse events within 4 days after RSVPreF3 OA or placebo administration (exposed population)**

|  | **Percentage of participants (95% CI)** | | | | |
| --- | --- | --- | --- | --- | --- |
| **Adverse event** | **50–59-non-AIR-RSV**  **N=377** | **50–59-non-AIR-placebo**  **N=191** | **50–59-AIR-RSV**  **N=379** | **50–59-AIR-placebo**  **N=188^a^** | **≥60-RSV**  **N=379** |
| **Administration-site adverse events** | | | | | |
| Erythema | 11.9 (8.8, 15.6) | 0.5 (0.0, 2.9) | 14.5 (11.1, 18.5) | 0.5 (0.0, 2.9) | 12.1 (9.0, 15.9) |
| Grade 3 | 0.0 (0.0, 1.0) | 0.0 (0.0, 1.9) | 1.1 (0.3, 2.7) | 0.0 (0.0, 1.9) | 0.8 (0.2, 2.3) |
| Pain | 76.4 (71.8, 80.6) | 10.5 (6.5, 15.7) | 75.2 (70.5, 79.5) | 13.8 (9.2, 19.6) | 61.2 (56.1, 66.1) |
| Grade 3 | 3.2 (1.7, 5.5) | 0.5 (0.0, 2.9) | 3.7 (2.0, 6.1) | 0.0 (0.0, 1.9) | 2.1 (0.9, 4.1) |
| Swelling | 9.3 (6.6, 12.7) | 1.0 (0.1, 3.7) | 11.6 (8.6, 15.3) | 0.5 (0.0, 2.9) | 7.7 (5.2, 10.8) |
| Grade 3 | 0.0 (0.0, 1.0) | 0.0 (0.0, 1.9) | 0.3 (0.0, 1.5) | 0.0 (0.0, 1.9) | 0.0 (0.0, 1.0) |
| **Systemic adverse events** | | | | | |
| Arthralgia | 26.0 (21.6, 30.7) | 5.8 (2.9, 10.1) | 20.8 (16.9, 25.3) | 10.1 (6.2, 15.3) | 12.9 (9.7, 16.7) |
| Grade 3 | 1.6 (0.6, 3.4) | 0.0 (0.0, 1.9) | 1.8 (0.7, 3.8) | 1.6 (0.3, 4.6) | 1.1 (0.3, 2.7) |
| Fatigue | 43.8 (38.7, 48.9) | 17.3 (12.2, 23.4) | 35.9 (31.0, 40.9) | 19.0 (13.7, 25.4) | 23.7 (19.6, 28.4) |
| Grade 3 | 3.4 (1.8, 5.8) | 0.5 (0.0, 2.9) | 2.1 (0.9, 4.1) | 1.1 (0.1, 3.8) | 1.8 (0.7, 3.8) |
| Fever | 3.7 (2.0, 6.2) | 1.0 (0.1, 3.7) | 2.6 (1.3, 4.8) | 1.1 (0.1, 3.8) | 1.6 (0.6, 3.4) |
| Grade 3 | 0.3 (0.0, 1.5) | 0.5 (0.0, 2.9) | 0.0 (0.0, 1.0) | 0.5 (0.0, 2.9) | 0.0 (0.0, 1.0) |
| Headache | 35.8 (31.0, 40.9) | 16.8 (11.8, 22.8) | 27.7 (23.3, 32.5) | 16.9 (11.9, 23.1) | 21.1 (17.1, 25.6) |
| Grade 3 | 3.4 (1.8, 5.8) | 1.0 (0.1, 3.7) | 1.8 (0.7, 3.8) | 1.1 (0.1, 3.8) | 0.8 (0.2, 2.3) |
| Myalgia | 39.0 (34.0, 44.1) | 5.8 (2.9, 10.1) | 32.2 (27.5, 37.2) | 13.8 (9.2, 19.5) | 21.1 (17.1, 25.6) |
| Grade 3 | 2.7 (1.3, 4.8) | 0.0 (0.0, 1.9) | 2.4 (1.1, 4.5) | 1.1 (0.1, 3.8) | 0.8 (0.2, 2.3) |

Fever was defined as a temperature ≥38.0°C. Grade 3 adverse events were defined as administration-site erythema or swelling with a diameter >100mm, fever with a temperature >39.0°C, and administration-site pain, headache, fatigue, myalgia, and arthralgia that prevented normal activity. 50–59-non-AIR-RSV/placebo, group of 50–59-year-old participants without increased risk for respiratory syncytial virus (RSV) disease who received RSV prefusion F protein-based vaccine (RSVPreF3 OA)/placebo; 50–59-AIR-RSV/placebo, group of 50–59-year-old participants at increased risk for RSV disease who received RSVPreF3 OA/placebo; ≥60-RSV, group of ≥60-year-old participants who received RSVPreF3 OA; N, number of participants with available results; CI, confidence interval.

^a^N=189 for systemic adverse events.

**Supplementary references**

1. Miettinen O, Nurminen M. Comparative analysis of two rates. Stat Med **1985**; 4:213-226.

2. Bretz F, Maurer W, Brannath W, Posch M. A graphical approach to sequentially rejective multiple test procedures. Stat Med **2009**; 28:586-604.
